# Beyond the Surface: Endocytosis of Mosquito-Borne Flaviviruses

**DOI:** 10.3390/v13010013

**Published:** 2020-12-23

**Authors:** Stephen D. Carro, Sara Cherry

**Affiliations:** Department of Pathology and Laboratory Medicine, Perelman School of Medicine, University of Pennsylvania, Philadelphia, PA 19104, USA; sdcarro@pennmedicine.upenn.edu

**Keywords:** flavivirus, mosquito-borne, clathrin-mediated, endocytosis, receptors, RNASEK, LY6E, entry inhibitor, nanchangmycin

## Abstract

Flaviviruses are a group of positive-sense RNA viruses that are primarily transmitted through arthropod vectors and are capable of causing a broad spectrum of diseases. Many of the flaviviruses that are pathogenic in humans are transmitted specifically through mosquito vectors. Over the past century, many mosquito-borne flavivirus infections have emerged and re-emerged, and are of global importance with hundreds of millions of infections occurring yearly. There is a need for novel, effective, and accessible vaccines and antivirals capable of inhibiting flavivirus infection and ameliorating disease. The development of therapeutics targeting viral entry has long been a goal of antiviral research, but most efforts are hindered by the lack of broad-spectrum potency or toxicities associated with on-target effects, since many host proteins necessary for viral entry are also essential for host cell biology. Mosquito-borne flaviviruses generally enter cells by clathrin-mediated endocytosis (CME), and recent studies suggest that a subset of these viruses can be internalized through a specialized form of CME that has additional dependencies distinct from canonical CME pathways, and antivirals targeting this pathway have been discovered. In this review, we discuss the role and contribution of endocytosis to mosquito-borne flavivirus entry as well as consider past and future efforts to target endocytosis for therapeutic interventions.

## 1. Mosquito-Borne Flaviviruses: Replication, Disease, and Epidemiology

Flaviviruses are positive-sense, single-stranded RNA viruses primarily transmitted by arthropod vectors, most notably mosquitoes and ticks [1,2]. The *Flavivirus* genus is generally broken into four subgroups: flaviviruses that are mosquito-borne, those that are tick-borne, those that infect only insects, and those without a known vector [3]. The mosquito-borne flavivirus subgroup is home to several transmissible human pathogens, including dengue virus (DENV), yellow fever virus (YFV), Zika virus (ZIKV), West Nile virus (WNV), and Japanese encephalitis virus (JEV). The impact of these flaviviruses is global, and there remains widespread infection and disease [4,5,6,7,8,9]. Although some vaccines exist, including for YFV and JEV, vaccine efficacy against DENV is limited, and there is limited production for these vaccines [10,11,12,13,14,15,16]. For other mosquito-borne flaviviruses where vaccines have not been developed or approved, there remains a large gap in therapeutic options. Indeed, there are no specific antivirals to treat these flaviviral infections. Though a variety of small molecules and inhibitors have been described to limit mosquito-borne flavivirus replication and disease in model systems, none have gone into clinical development. Given the global spread of flavivirus infection and the significant public health threat of emerging viruses, there remains a critical need for further flavivirus research [17].

### 1.1. Flavivirus Life Cycle

To begin an infection, the virion must attach to the surface of the target cell [18]. There are two classes of receptors that the virus can interact with: attachment factors that increase surface binding, and true receptors that are required for internalization. Attachment factors can bind to diverse molecules including phosphatidylserine on the viral lipid bilayer or heparan sulfate and other sugars on the viral glycoprotein E. Next, most flaviviruses require endocytosis to traffic into a low pH compartment for fusion. In most cases, the pathway used by flaviviruses is clathrin-dependent uptake. These vesicles are trafficked into a low pH endosomal compartment where the viral E protein enters into a fusion-active state, permitting fusion between the endosomal membrane and viral envelope. The fusion process releases the single-stranded, positive-sense RNA genome into the host cytoplasm, which is translated by the host into a polyprotein and subsequently cleaved into three structural and seven non-structural proteins. Following genome replication and immature virion assembly within virus-induced and endoplasmic reticulum (ER)-derived replication complexes, viral particles bud into the ER and are trafficked through the trans-Golgi network, undergoing maturation through proteolytic cleavage events and pH changes. Upon reaching the cell surface, particles are exocytosed and the infection cycle can begin anew.

### 1.2. Dengue Virus (DENV)

Dengue virus (DENV) is the most widespread of the pathogenic flaviviruses, exhibiting transmission throughout the tropics and sub-tropics [19]. It is estimated that 400 million DENV infections occur globally every year, with the majority of those being sub-clinical infections (i.e., asymptomatic or mild) [19]. DENV transmission is largely maintained through an urban cycle between humans and Aedes mosquitoes, but there remains a sylvatic cycle with nonhuman primates [20]. Regular descriptions of dengue-like disease first emerged in the 18th century across numerous continents, though there are scattered reports dating as far back as 992 CE [21]. In the mid-20th century, initiated by World War II, urbanization, globalization, and a concurrent lack of vector control permitted the widespread expansion of DENV [22]. Unsurprisingly, as Aedes vectors spread, so did the virus [23]. There are four serotypes of DENV (DENV-1, -2, -3, and -4) with each serotype being genetically distinct, and antigenic variation within serotypes is broad [21,24]. Currently, all four serotypes are co-circulating globally. Clinically, DENV disease presents along a continuum, ranging from asymptomatic disease to severe vascular permeability with high risk of mortality [25]. Infection with one serotype of DENV confers lifelong immunity to that serotype, but a subsequent, secondary infection by a heterotypic serotype has been associated with an enhanced risk for severe dengue disease, thought to be mediated by antibody-dependent enhancement [26,27,28]. Generally, it is thought that antibodies that bind but do not neutralize the newly encountered viral serotype can lead to enhancement of secondary infection through Fc receptor engagement [29]. Secondary infections are largely responsible for severe dengue in children and adults; however, infants can experience severe disease from a primary infection potentially as a result of maternally-acquired enhancing antibodies [30]. Antibody-dependent enhancement is a major risk factor with serious implications in DENV vaccine design [10,11,12]. In addition, current DENV control efforts include vector control programs [31].

### 1.3. Yellow Fever Virus (YFV)

Yellow fever virus (YFV) causes what many believe to be the original viral hemorrhagic fever, yellow fever, which manifests as a systemic illness leading to fever, jaundice, hepatic injury, renal injury, and hemorrhage, with case fatality rates of ~30% [32,33,34]. Though YFV is an ancient virus, showing molecular divergence as far as 3000 years ago [35], the virus entered the Americas 300–400 years ago through the slave trade with Africa [36]. After its introduction into the New World, YFV caused a series of ravaging epidemics across various seaports prior to the turn of the 20th century [37,38]. During the early- to mid-20th century, a vaccine (YFV-17D) was developed through continuous passaging of virus in vitro, ultimately providing a means to prevent yellow fever and leading to a significant reduction in cases [39]. This is the same vaccine that is used today. Vector eradication ultimately blocked the YFV transmission cycle, especially in the Americas, though the discontinuation of early efforts has permitted the reemergence of vector populations [40,41]. In the modern day, YFV still persists, and its geographical distribution largely resembles that of its vectors (Aedes, Haemagogus, and Sabethes mosquitoes) [41]. Africa has a YFV endemic region that spans 15° north and south of the Equator [41,42], with heterogeneity in geographical and temporal disease burden attributed to seasonality as well as differences in YFV genotypes [43]. Severe cases on the scale of hundreds of thousands are estimated to occur every year in Africa [44]. Outside of Africa, YFV is endemic to many regions across South America [42], and recent epidemics in South America have re-highlighted the capacity for YFV to effectively spread and re-establish in previously YFV-free areas [34,45,46,47,48]. In endemic regions, vaccination programs can manage case numbers, but global supplies are often not sufficient [49]. Even with widespread vaccination, YFV could not be eradicated, as the virus is well established in sylvatic transmission cycles between mosquito vectors and non-human primates [41,48]. Continued surveillance for YFV is essential for the prevention of future epidemics, especially considering the spread of vector populations into non-endemic regions [23,48].

### 1.4. Zika Virus (ZIKV)

Zika virus (ZIKV) was first discovered in the mid-20th century, but the virus did not garner attention until its emergence on the Micronesian island of Yap in 2007 [50,51,52]. In the following decade, ZIKV re-emerged in a series of epidemics, most notably in French Polynesia from 2013 to 2014 [53] and in the Americas from 2015 to 2016 [9,54], which cast a light on the pathogenesis, clinical manifestations, and outcomes of Zika virus disease. Early findings from French Polynesia indicated that the typical length of Zika-induced disease was one week, with most symptomatic patients exhibiting rash, fatigue, fever, arthralgia, myalgia, or conjunctivitis [51,53]. However, there are a variety of additional and more severe complications described to be in association with ZIKV infection, including Guillain-Barré syndrome [55], myelitis [56], meningoencephalitis [57], and thrombocytopenia [58]. ZIKV has a potential for transmission in the absence of the typical Aedes vector, including both vertical and sexual transmission. Sexual transmission is well-documented and thought to be largely driven by the persistence of infectious virus within semen [59,60,61,62,63]. On its own, sexual transmission does not pose a major threat to populations. Sexual transmission can, however, contribute to vertical transmission of ZIKV, which bears more serious consequences. Unlike in children and adults, where most ZIKV infections are asymptomatic or very mild, ZIKV can lead to a plethora of fetal defects and complications associated as a result of maternal ZIKV infection and subsequent vertical transmission. These manifestations include microcephaly, cerebral malformations, hydrops fetalis, and fetal demise [64,65,66,67,68,69,70]. A variety of laboratory models substantiated these clinical findings [71,72,73]. 

ZIKV has a very high attack rate in populations, leading to rapid acquisition of herd immunity. Therefore, the prevalence of ZIKV has drastically declined since the epidemic in 2016 in the Americas [74]. However, the virus remains a public health concern, especially considering the lack of approved therapeutics, the clinical manifestations of disease, and the likelihood that the virus will re-emerge once population immunity has declined [9,54,75]. Future efforts must be focused towards vaccine deployment in combination with diagnostic surveillance and identification of key ecological and social risk factors that may promote virus spread [76].

### 1.5. West Nile Virus (WNV)

West Nile virus (WNV) was first discovered in the West Nile district of the Ugandan Northern region in 1937 [77]. Up until the late 20th century, WNV disease was infrequent and mild, sporadically arising across Africa, Asia, and Europe. However, a series of European outbreaks in the 1990s changed the outlook of WNV infection [78]. For example, in 1996, an epidemic of WNV disease occurred in Romania, resulting in hundreds of infected patients, the majority of which presented with central nervous system symptoms [79]. In 1999, WNV spread to the US and was first found in New York City when there was a dramatic increase in dead birds and encephalitis cases [80,81]. Early diagnoses suggested that St. Louis encephalitis virus (SLEV), another neurotropic flavivirus, was the causative agent, but the concurrent encephalitic death of avian species housed in the Bronx zoo and those in the surrounding geographic area led to the discovery of WNV [81,82]. The 1999 US WNV outbreak was originally traced back to Israel [82,83,84]. Surprisingly, within a few years, WNV rapidly expanded across the United States and into several Canadian provinces, Mexico, the Caribbean islands, and South America, with as many as 3 million estimated infections by 2010 in the United States alone [85,86]. Since the late 20th century, WNV has established itself as a new endemic threat in all continents besides Antarctica [6,17,87,88,89,90]. In nature, WNV is maintained by an enzootic transmission cycle between Culex mosquitoes and a variety of birds, with the virus exhibiting broad host range [90]. Most mammals, including humans, serve as dead-end hosts in the WNV transmission cycle [91,92].

### 1.6. Japanese Encephalitis Virus (JEV)

Following reports of encephalitis epidemics in Japan throughout the 19th and early 20th centuries, Japanese encephalitis virus (JEV) was isolated in 1935, demonstrating its differences from the clinically similar Saint Louis encephalitis virus [93]. As with many flaviviruses, molecular divergence of JEV from its ancestor is estimated to have occurred more than 3000 years ago [94,95]. Currently, there are five known genotypes of JEV (I-V), and each genotype is associated with particular geographic locations and climatic conditions [94,96,97,98,99,100]. JEV has historically remained endemic throughout east and southeast Asia [101]. However, more recently, the virus underwent geographic expansion, as evidenced by reports of isolated cases or outbreaks in Pakistan [102], Australasia [103,104,105], and even Africa [106]. This highlights JEV as an emerging threat, and as much as half of the world’s population lives in a JEV endemic country. Moreover, there is concern that the number of people at risk may grow larger if the virus continues to spread globally, including to the Americas, similar to WNV [107,108]. Although there exist several effective and potentially cross-protective vaccine options, underuse, cost, lack of supply, and unavailability have limited their utility [107,109,110,111,112]. As many as 50,000 cases of disease and 15,000 deaths occur on a yearly basis as a result of JEV infection, demonstrating a distinct need for more widespread protection [113]. Clinically, the majority of JEV infections result in a mild non-specific or asymptomatic disease [114]. Those with more severe symptoms can present with convulsions, seizures, flaccid paralysis, meningitis, and encephalitis, with half of surviving patients having severe neurological sequelae [114,115]. In nature, JEV transmission is maintained by an enzootic cycle between Culex mosquitoes, wading birds, and pigs, with humans serving primarily as dead-end hosts [116]. However, recent considerations have suggested that the transmission cycle of JEV is largely context dependent, partially varying between geographic regions [117].

## 2. Mosquito-Borne Flavivirus Receptors

Across the mosquito-borne flaviviruses, a wide array of attachment factors and receptors have been implicated in viral entry. Generally, the purposes of attachment factors are to increase the binding of the virions to the cell surface and to facilitate interactions with viral receptors, which are required for infection. Attachment factors typically increase the affinity of the virus for the target cell but are dispensable for infection while the bone fide receptor is essential for viral infection. The identification of receptors for mosquito-borne flaviviruses, however, has proven to be difficult, and many candidates often blur the distinction between attachment factor and host receptor.

The TAM subfamily of receptor tyrosine kinases (RTKs), which consists of three members, Tyro3, Axl, and Mertk, has been implicated in the attachment of diverse flaviviruses [118,119]. Axl was first suggested to be a flaviviral entry factor in the context of DENV infection, where Axl, along with its cognate ligand, Gas6, was shown to be permissive of DENV entry into HEK293 cells through indirect DENV-Gas6-Axl binding [120]. In contrast, Axl, along with its TAM family member, Mertk, was implicated in protection from neuroinvasive WNV infection by maintaining blood–brain barrier (BBB) integrity, a finding that suggests context-specific roles for Axl in flavivirus infection [121]. In the context of ZIKV infection, initial studies showed that Axl promoted ZIKV internalization in HEK293T cells, and Axl surface expression positively correlated with ZIKV infection of A549 lung epithelial and HFF1 fibroblast cell lines [122]. The alternative TAM receptor, Tyro3, as well as the C-type lectin receptor, DC-SIGN, also promoted ZIKV infection in HEK293T cells [122]. The role of Axl as a flavivirus entry factor was further supported in ZIKV infection of diverse cell types, including human endothelial cells [123,124,125]. Studies aiming to establish routes of ZIKV infection into fetal brains found that ZIKV was able to infect iPSC-derived neural progenitor cells (NPCs), resulting in attenuated growth and cell-cycle dysregulation [126]; high levels of Axl were also observed in brain cell populations [127,128]. However, subsequent experiments in iPSC-derived NPCs and cerebral organoids discovered that Axl null mutants remain susceptible to ZIKV [129], and mouse models deficient for Axl as well as its TAM family relatives, Mertk and Tyro3, remain susceptible across various anatomical sites, including the brain [130,131,132]. These results suggest that Axl does not have a definitive role in ZIKV entry and may play a more significant role in other facets of biology, including immune modulation. In diverse studies, treatment with Axl inhibitors blocked infection, but only in cells that express the receptor, again suggesting that Axl is a dispensable factor for entry [133,134,135]. Taken together, Axl may serve as an attachment factor in certain cell types, but the possibility remains likely that the TAM receptors primarily impact innate immunity. The downregulation of innate immune responses by TAM receptors during viral infection has been established [136,137].

Integrins have been implicated in attachment and entry of several flaviviruses, including JEV [138], WNV [139,140], and ZIKV [141], alongside other transmembrane proteins such as TIM-1, which has been described to facilitate DENV infection [142]. In addition, a series of genome-wide CRISPR-Cas9 screens have been performed to identify other host factors required for flavivirus infection [143,144,145,146,147]. None have defined a definitive receptor. There are several possibilities to account for this lack of definitive receptor discovery. First, there may be cell-type-specific entry receptors; indeed, many entry factors are cell-type specific. Second, there may be multiple receptors and thus single gene efforts would miss other possible candidates. Therefore, it is important to investigate viral entry in biologically relevant systems using a variety of approaches with stringent downstream analysis to validate candidate entry factors.

## 3. Endocytic Pathways Mediating Mosquito-Borne Flavivirus Internalization

### 3.1. Clathrin-Mediated Endocytosis

Many flaviviruses are internalized by clathrin-mediated endocytosis (CME). Following internalization, viruses traffic to an endosomal compartment where the low-pH environment induces uncoating and fusion of the viral envelope with the endosomal membrane, ultimately allowing the deposition of viral RNA into the host cytoplasm [18].

The process of CME is highly regulated and exhibits modular behavior, with a series of steps dependent on specific complexes, which dynamically assemble and disassemble the clathrin-coated vesicles at distinct time points [148]. This process requires two primary classes of proteins: adapter and scaffold proteins. The clathrin-adapter proteins (e.g., AP2) associate with and bind lipids within the plasma membrane as well as the various cargo molecules. Scaffold proteins, including clathrin, establish intra- and intermolecular interactions with themselves as well as with the clathrin-adapter proteins, respectively. Clathrin proteins consist of three heavy chain polypeptides each in association with a light chain polypeptide to form what is known as a triskelion [149]. At the amino terminal ‘tip’ of each heavy chain is the terminal domain, which is important for establishing interactions with various adapter proteins, such as the AP2 complex. These clathrin triskelions assemble around a membrane invagination as a lattice, which is the final structure of the clathrin coat. Upon release of a clathrin-coated vesicle, uncoating is mediated by interactions between proteins such as Hsc70 and the carboxy terminus of the clathrin heavy chains.

**Figure 1:**
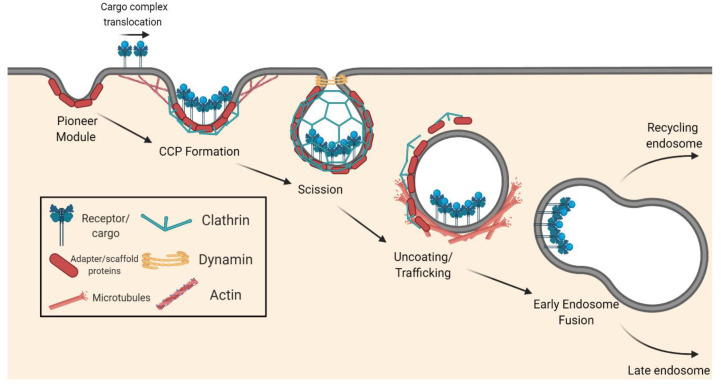
Schematic of the canonical clathrin-mediated endocytosis (CME) pathway. Initially, a group of adapter and scaffold proteins associate at the endocytic site, forming a pioneer module. This pioneer module then recruits coat-associated proteins, including clathrin, to the developing membrane invagination. The coat-associated proteins bind to the cytosolic regions of cargo complexes to recruit the cargo to the endocytic site. Membrane bending continues, in part due to mechanical forces from actin, and the clathrin coat expands until the clathrin-coated pit (CCP) is formed. Scission of the CCP is mediated by dynamin, which releases the clathrin-coated vesicle (CCV). The CCV then uncoats and undergoes trafficking via the cytoskeleton to the sorting endosome for further processing. Figure created with BioRender.com.

The first step of CME involves initiation of the pit and assembly of a clathrin coat around a membrane invagination (Figure 1). A group of early proteins known as the “pioneer module”, which includes the BAR domain proteins FCHO1/2, the AP2 complex, ESP15, EPS15R, and intersectins, is thought to be the primary initiator of endocytosis in mammals [148]. FCHO1/2 and AP2, as adapters, bind to the plasma membrane and are responsible for the recruitment of the scaffold proteins (EPS15, EPS15R, and intersectins). This protein complex defines the endocytic site, and shortly after its formation, additional coat-associated proteins, including clathrin, are recruited to the plasma membrane to expand the coat.

During the second step, the various coat-associated proteins bind to the cytosolic regions of transmembrane cargo receptors via sequence motifs or covalent modifications and act as cargo adapters to induce translocation of the cargo complexes to the vesicle-forming region of the plasma membrane. In association with cargo, the clathrin coat continues to dynamically expand, which induces membrane bending and results in the formation of a clathrin-coated pit (CCP). Actin polymerization transiently contributes to membrane bending, where actin filaments are coupled to the clathrin coat via adapter proteins in order to exert mechanical force upon the plasma membrane.

Third, after the maturation of the membrane invagination, vesicle scission is mediated by the GTPase dynamin, ultimately separating the CCP from the donor membrane. The resulting clathrin-coated vesicles (CCVs) are typically 700–800 Å in diameter, contain about 35–40 triskelions, and enclose a spherical vesicle ~400 Å in diameter, but they can vary in both diameter and number of clathrin triskelion components [149]. Lastly, the vesicle coat disassembles, and the components are recycled for additional endocytic events. This allows the free vesicle to traffic to the early endosome for sorting. From there, vesicles can be trafficked to various compartments, initiating different intracellular fates dependent on protein families such as the Rab proteins [150].

Each endosomal compartment has key characteristics, including luminal pH, phosphatidylinositol lipids, and association with Rab-family GTPases. The early/sorting endosome is enriched for PI(3)P and specifically involves the activation of the Rab5 GTPase [150]. From here, cargo can be recycled back to the plasma membrane through the Rab11-positive recycling endosome, or be trafficked to the PI(3,5)P_2_-enriched, Rab7-positive late endosome. Unlike in the early endosomes, where luminal pH is maintained around 6.5, the late endosome has a luminal pH of ~5.5. Some viruses fuse within early endosomes while others fuse in later compartments, and this is dependent on the pH requirement for fusion. From the late endosome, cargo can either be sent to the trans-Golgi network for retrograde trafficking or sorted to the lysosome for degradation.

Although CME has been well-studied over the years, the exact molecular mechanisms driving vesicle invagination and scission remain unclear [148]. Additionally, the regulatory complexities of CME are ever-growing, and increasing evidence suggests that cargo-adapter complexes strongly influence early steps of CME, whether through the identity of the complex itself or the identities of interacting partners [148,151]. Considering the diversity of endocytic cargo (e.g., protein ligands versus whole viral particles), it is not surprising that there is a high level of regulation. Though it has been long known that flaviviruses undergo CME during host cell infection, recent evidence suggests that entry mechanisms of flaviviruses depend on a non-canonical, specialized form of CME [152,153].

### 3.2. Flaviviruses and Clathrin-Mediated Endocytosis

For several decades, it has been known that flaviviruses enter host cells via an endocytic route. Early studies investigating electron micrographs of WNV-infected cells identified endocytosis as the primary WNV entry pathway [154]. These images showed viral particles within coated invaginations on the plasma membrane and eventually within coated and uncoated vesicles in the cytosol. Later time points revealed localization of viral particles within electron-lucent pre-lysosomal vesicles. Key cellular players in endocytic WNV entry, including clathrin and EPS15, were identified initially in mosquito cells through pharmacological inhibitors, blocking antibodies, and dominant negative constructs, highlighting similarities in endocytic pathways in vertebrate and vector-derived cell lines [155,156]. Additional studies also found that many flaviviruses use similar internalization pathways. For example, electron micrographs of JEV infection into the CNS showed the involvement of coated endocytic vesicles [157], as did electron micrographs of YFV 17D [158] and WNV-Kun [159]. Each of these studies found virions in coated, endocytic vesicles during viral entry. Live-cell imaging and viral tracking revealed a near exclusive use of CME for viral entry of DENV-2, where as many as 92% of viral particles were shown to diffuse along the cell surface, colocalize with a pre-existing CCP, undergo internalization and eventually complete membrane fusion [160]. While some studies suggest direct fusion of DENV-2 at the plasma membrane [161,162], the vast majority of studies suggest that CME is required [163,164,165,166,167,168,169].

In contrast, some studies have suggested that non-CME endocytic routes can be used by flaviviruses. In YFV infection of HeLa cells, for example, the vaccine strain 17D was shown to use a clathrin-independent mode of entry while the wild-type Asibi strain exhibited the classical dependence on clathrin-mediated endocytosis [170]. DENV-2 NGC and DENV-3 H87 infection of Vero cells has been previously reported to occur via a clathrin-independent but dynamin-dependent mechanism [171,172]. In general, ZIKV appears to be dependent on CME across divergent strains in diverse cell types [173,174], although a study in neural cells suggested a weak dependence [146]. Similarly, JEV was reported to infect neuronal cell lineages via a clathrin-independent pathway [175,176,177] in contrast to the clathrin-dependent endocytic mechanisms displayed in Vero, C6/36, BHK-21, HeLa and PK15 cells [178,179,180,181,182]. Whether neurons in general have distinct trafficking requirements has yet to be established more generally. In addition, the glycosylation of viruses can differ with the cell types used to produce virus, which can lead to differences in entry. This is particularly important when studying arboviruses that are produced in both mosquito and vertebrate cells in nature. For example, cell type-dependent effects on entry have been described for DENV-2 grown in primate cells versus mosquito cells [183].

Live cell imaging of DENV-2 also found that after internalization, the majority of viral particles entered Rab5-positive early endosomes, and the compartment either fused with an existing Rab7-positive late endosome or matured into a late endosome prior to viral membrane fusion [160]. Moreover, other studies found that Rab5 but not Rab7 was important during CME of DENV-2 [184]. Indeed, different strains of DENV may require different pH thresholds for membrane fusion, leading to different trafficking dependencies observed between DENV-2 strains [160].

Altogether, while most studies found a role for CME in viral entry of diverse flaviviruses, it is likely that there is variability at the level of strain, serotype, and cell type. Whether these variations translate in vivo is unclear, but these findings suggest that there may be alternative entry pathways used in vivo. Recent efforts have found new factors involved in flavivirus entry, offering new mechanistic insights and avenues for antiviral development.

### 3.3. Flavivirus Entry: Beyond Traditional Clathrin-Mediated Endocytosis

To identify new players involved in viral entry and infection, many investigators have performed high-throughput screens. Genetic screens including RNAi, overexpression, and CRISPR/Cas9 screens have revealed host dependency factors that are necessary for flaviviral infection in diverse cell types and hosts. Subsequent studies have linked many of these newly identified genes to entry. A genome-wide siRNA screen in HeLa cells during WNV infection revealed a role for the E3 ubiquitin ligase, CBLL1, in virus internalization, with the knockdown phenotype resembling that induced by ablation of AP3S2, a clathrin adapter necessary for CME [185]. LY6E, a GPI-anchored surface protein, was also identified in multiple genetic screens as promoting flavivirus infection [185,186,187,188]. LY6E is a member of the Ly6 family of proteins, and the protein has been previously referred to as thymic shared antigen-1 (TSA-1) or stem cell antigen-2 (SCA-2). Early studies described roles for LY6E in diverse T cell receptor-mediated processes, such as modulation of apoptosis [189] and IL-2 production [190,191]. Only recently has its role in viral infection been elucidated. Initial studies demonstrated that LY6E knockdown does not impact the replication of a WNV replicon in HEK293T cells [153]. Viral binding and internalization assays with WNV-Kun specifically implicated LY6E function in virus internalization [153]. Parallel studies confirmed the infection-enhancing and post-attachment, pre-replication function of LY6E during YFV infection [192,193]. Interestingly, LY6E was shown to be dispensable for cellular uptake of canonical CME cargo, such as transferrin (~6 nm diameter), but necessary for internalization of virions, which are larger (~50 nm diameter) [153]. Indeed, it was shown that while transferrin was internalized independent of LY6E, transferrin-coated 40-nm microspheres were dependent on this factor, identifying a size-dependent step in CME [153]. Internalization of these larger cargo, including the microspheres, WNV-Kun, and ZIKV MR766 virions, induced the relocalization and restructuring of LY6E molecules into tubular structures, a process dependent upon microtubule function as well as the microtubule end-binding protein, EB3 [153]. GPI-anchored proteins are capable of regulating and interacting with components of the cytoskeleton more generally [194,195]. In light of these findings, LY6E appears to play a key role in a specialized CME pathway, one specifically intended for the internalization of large cargoes, such as flaviviruses. A role for LY6E in viral fusion is supported by recent findings implicating LY6E in syncytiotrophoblast layer formation [196,197]. LY6E may also enhance viral membrane fusion through modulation of membrane lipids, perhaps altering lipid raft dynamics [188]. Future studies are required in order to fully elucidate the molecular mechanisms mediating LY6E function in the context of viral infection.

Additional genetic screens identified a role for RNASEK, a small membrane-spanning protein with unclear function, in CME and entry of viruses including flaviviruses [152,198,199]. Initially identified in RNAi screens in both Drosophila cells [200,201] and human HeLa cells [198], RNASEK depletion was shown to inhibit infection of human rhinovirus (HRV), DENV2/3/4, YFV 17D, WNV-Kun, Rift Valley fever virus (RVFV), Sindbis virus (SINV), and influenza A virus (IAV) across various cell types [152,198]. Attachment and internalization assays defined a role for RNASEK in virus internalization and CME [152,198]. Furthermore, it was found that RNASEK was required to maintain proper V-ATPase function, possibly through physical association [198]. The V-ATPase is a proton pump responsible for acidifying intracellular compartments and a regulator of clathrin-coated vesicle formation [199,202,203]. Recent cryo-EM images of the V-ATPase support its physical association with RNASEK, suggesting that RNASEK is a member of this protein complex [204]. There are data that suggest that the V-ATPase is present both on the plasma membrane and intracellular endosomes [205], and there is support for RNASEK function in both compartments. In addition to attenuation of endocytic uptake, loss of the V-ATPase or RNASEK in H1-HeLa cells resulted in increased intracellular acidity and an enlargement of clathrin-coated pit (CCP) size [198]. These enlarged CCPs exhibited a higher incidence of tethering, suggesting that RNASEK contributes to steps upstream of dynamin-mediated scission [198]. Confocal imaging revealed that RNASEK and V-ATPase subunit protein signals originate near the inner leaflet of the plasma membrane (i.e., towards the base of the CCP) and significantly colocalize with clathrin-adapter proteins [198]. Interestingly, RNASEK appeared to be necessary for the localization of V-ATPase subunits at the base of the CCP (near the plasma membrane), offering a role for RNASEK in V-ATPase targeting [198]. Loss of RNASEK, however, did not reduce colocalization of general CME-associated factors at the CCP, suggesting that the contribution of RNASEK to membrane localization is specific for the V-ATPase [198]. These imaging studies argue for a localization of RNASEK and the V-ATPase either at or in close association with the plasma membrane. 

There is some discrepancy on the dependence of classical endocytosis on RNASEK: in H1-HeLa cells, RNASEK was necessary for canonical CME (i.e., endocytosis of transferrin) [198], but in U2OS cells, RNASEK was only essential for non-canonical cargo uptake (e.g., virus particles) and was dispensable for smaller cargo, such as transferrin [152]. Follow-up investigations in U2OS cells integrated the role of RNASEK into the LY6E-mediated size-dependent CME pathway, where the tubularization of LY6E induced by flavivirus uptake is hindered in the absence of RNASEK [153]. Such a result suggests that RNASEK may act upstream of LY6E, or at least upstream of LY6E tubularization. There remain numerous questions regarding the specific function of RNASEK in CME and how the V-ATPase impacts RNASEK function. It is also not fully clear how the V-ATPase regulates CME. In a genome-wide siRNA screen for regulators of CCV formation, knockdown of the V-ATPase resulted in the formation of large and irregular CCPs at the plasma membrane [199], with the pits being unable to constrict and form CCVs, as with RNASEK knockdown [198]. This phenotype was attributed to intracellular accumulation and improper recycling of cholesterol, likely due to alkalization of endosomes, which could be partially rescued through the addition of exogenous cholesterol [199]. In contrast, the importance of cholesterol in CME regulation was not supported in the studies of RNASEK [198]; therefore, how cholesterol and other lipids impact CME and virus entry remains incompletely understood.

**Figure 2:**
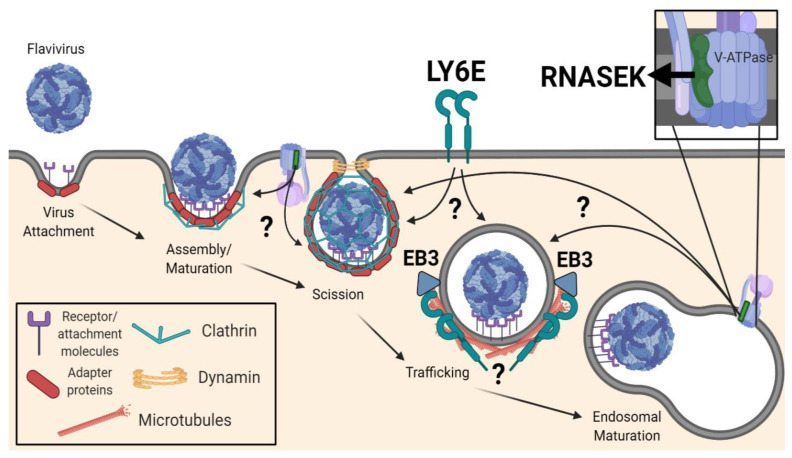
Schematic of the proposed size-dependent clathrin-mediated endocytic (CME) pathway. Following initial binding of an incoming flavivirus virion to the cell membrane, virions can bind attachment factors and receptors. Next, internalization begins and a clathrin coat forms around the membrane invagination via adapter molecules. Scission of the clathrin-coated vesicle is mediated by the GTPase dynamin. The internalized virion then traffics to the endosomal compartment for acid-mediated membrane fusion. During the internalization process, the GPI-anchored LY6E and V-ATPase-associated RNASEK perform essential roles outside of the canonical CME pathway. LY6E tubularization is induced by viral entry, a process dependent on microtubule function, the end-binding protein EB3, and RNASEK. Figure created with BioRender.com.

A specialized size-dependent endocytic uptake pathway distinct from canonical CME has begun to be characterized for flaviviruses (Figure 2) [152,153]. However, there remain numerous questions about the complexities and the prevalence of this pathway. How newly identified players, such as LY6E and RNASEK, interact with canonical CME proteins, in what contexts they are required, and at what steps they contribute still remains unclear, necessitating additional studies. The seemingly disparate dependence on RNASEK for canonical CME may be variable given the cell type. It also remains possible that the contribution of the V-ATPase to CME via endosomal cholesterol trafficking [199] is separable from the function of RNASEK and V-ATPase at the plasma membrane [198]. However, the lack of rescue by exogenous cholesterol in the enlarged CCP phenotype induced by the depletion of RNASEK or V-ATPase in H1-HeLa cells argues for alternative mechanisms [198]. Additionally, whether or not RNASEK and the V-ATPase are in direct association with the plasma membrane is unsolved. One can imagine a scenario where the endosome is trafficked, possibly through the action of RNASEK, towards the plasma membrane, allowing for close but not integral association between the plasma membrane and RNASEK and the V-ATPase. This potential trafficking towards the plasma membrane could be related to the dependence on microtubules for size-dependent CME [153], which is supported by the well-known link between endosomal motility and the cytoskeleton [206]. Alternatively, RNASEK may be in association with a specific isoform of the V-ATPase, one that localizes within the plasma membrane to exert its function. Focusing future work towards identifying other key molecules within this distinct pathway and subsequently defining how these factors interact with one another will likely aid in elucidating the molecular mechanisms of RNASEK, the V-ATPase, and LY6E in size-dependent CME. As our understanding of this potentially virus-specific CME pathway evolves, we can begin to evaluate the therapeutic targetability of this pathway as an avenue for antiviral treatments. Further studies may also be able to tie ‘miscellaneous’ flavivirus entry factors and their roles, if any, into this specialized CME pathway, including GPCR kinase, GRIK2 [207], and the endoplasmic reticulum membrane complex (EMC) [143]. Overall, enhanced definition of the molecular mechanism driving size-dependent entry and its prevalence across cell types and viral infections will help in the evaluation of its biological and in vivo significance, both in the context of antiviral therapeutics as well as the fundamental cell biology of CME.

## 4. Targeting Endocytic Entry: An Avenue for Flavivirus Antivirals

A broad series of flavivirus antiviral screens have identified diverse candidates, many of which have unknown targets but appear to impact early stages of viral infection (see Table 1). Additionally, a variety of FDA-approved drugs have been described to impact viral endocytosis. Given that CME is essential, drugs that inhibit the pathway can block infection but are typically toxic. These include inhibitors of clathrin or its adapters, such as chlorpromazine. Chlorpromazine is an FDA-approved medication historically used as an antipsychotic to treat a variety of mental illnesses through antagonism of dopamine D2 receptors (D2Rs) [208], though there is not a consensus among mechanisms of action [209]. Subsequent cellular studies found that chlorpromazine additionally blocks AP-2-plasma membrane binding, inhibiting the process of CME [210]. Therefore, in vitro chlorpromazine has been routinely used to demonstrate whether a virus is dependent on CME for entry [156,164,166,168,171,175,211,212,213,214]. The antiviral efficacy of chlorpromazine has been explored and demonstrated across various virus families [215,216,217,218], and a drug repurposing trial has even been proposed for SARS-CoV-2 [219]. Prochlorperazine, a structural analog of chlorpromazine, is also being considered as an antiviral [220]. Though the in vitro efficacy of chlorpromazine and prochlorperazine against flaviviral infection may be a result of their activities in inhibiting CME, there is increasing evidence that dopamine receptors themselves, and their associated signaling pathways, contribute to neurological flaviviral infections. Therefore, the antagonism of dopamine receptors may be the more important on-target effect that is driving antiviral efficacy. In DENV-2 infection, investigators have demonstrated that both infection of N18 neuroblastoma cells and the antiviral activity of prochlorperazine were dependent on the expression of D2R [220]. Similar dependencies were shown in Neuro-2a cells, where a D2R antagonist was capable of limiting DENV-2 infection and growth [221]. Downstream of D2R receptors, dopamine signaling has been suggested to actively contribute to and enhance infection of flaviviruses. In JEV infection of BE(2)C neuroblastoma cells, infection initiated dopamine production and release, which increased the susceptibility of other cells to JEV by increasing surface expression of entry factors [222]. Alternative dopamine receptors, such as D4R, have also been implicated in flavivirus infection [223]. Given that many dopamine receptor antagonists are already FDA-approved, repurposing of these drugs may prove to be an effective strategy for flavivirus antiviral development. 

Additional inhibitors of general CME have been developed and have shown antiviral properties, including dynasore [255], pitstops [253], and apilimod [256]. Dynasore is a noncompetitive inhibitor of the GTPase dynamin, which is required for scission of CCPs, and thus leads to the accumulation of U-shaped and O-shaped CCPs that have not yet pinched off [255]. Dynasore inhibits endocytosis of both canonical cargo, such as transferrin [255], and non-canonical cargo, such as viruses [168,171,175,213]. The pitstops (pitstop-1 and pitstop-2) are two small molecules that selectively target the terminal domain of clathrin, which is important for interactions between clathrin and adapter or accessory proteins during CME [253]. Treatment results in an increased lifetime of clathrin coat components and reversible inhibition of endocytosis for both canonical [253] and non-canonical cargo [250,252,253]. Unlike dynasore and pitstops, which target steps in CME prior to vesicle scission, apilimod is a small molecule that selectively targets the PI(3)P-5-kinase (PIKfyve), a lipid kinase present on the cytosolic face of endosomal membranes that is responsible for the generation of PI(3,5)P2 from PI(3)P [257]. PIKfyve is a key regulator of endosomal trafficking [258,259], and its inhibition by apilimod has been shown to be sufficient to prevent the entry of several viruses, presumably by limiting trafficking to the endosomal compartments necessary for viral fusion [227,228,229,230]. Apilimod has been clinically evaluated in the context of other diseases and presented with minor adverse side effects [260,261,262], though it was not shown to be an effective therapeutic in these contexts. Given its newly established role as an antiviral, the small molecule may find clinical use in the future. 

A variety of novel flavivirus antivirals have been recently described and specifically implicated in post-attachment entry. Several of these small molecules, including several flavonoid derivatives [244,254,263], stem from natural plant products and were tested in response to the recent 2015-2016 ZIKV epidemic [264]. Many natural products also demonstrated antiviral efficacy against other flavivirus members, including DENV and WNV [265,266,267,268]. In addition, niclosamide [245], tyrphostin A9 [245], BP34610 [234], and nanchangmycin [133] have been shown to have antiviral activities. Niclosamide, an anti-parasitic drug [247], and tyrphostin A9, an RTK inhibitor [269], were identified as the most potent broad-spectrum compounds in a screen for Semliki Forest virus (SFV) and DENV-2 antivirals that act at the step of virus entry [245]. Both drugs demonstrated antiviral efficacy against DENV-1, DENV-3, ZIKV, and YFV 17D as well, and in addition to blocking entry, they block internalization of transferrin [245]. Tyrphostin A9 may impact cargo-receptor signaling during endocytosis based on the mechanisms of chemical analogues [270] while niclosamide appears to impact endosomal acidification [246,247], though these proposed mechanisms are not definitive [245]. Another DENV screen identified BP34610, a candidate with antiviral efficacy against DENV-1/2/3/4 as well as JEV [234]. Time-of-addition assays revealed a role for BP34610 in viral entry, and resistant viruses suggested the compound likely targets the virion, rather than a host pathway.

A high-throughput screen against ZIKV identified the polyether antibiotic nanchangmycin as a potent inhibitor of infection for flaviviruses including ZIKV, DENV and WNV across various cell types [133]. Nanchangmycin blocked an early step during viral endocytosis, but the small molecule did not demonstrate inhibitory effects on CME of the canonical cargo transferrin. However, nanchangmycin blocked the CME of transferrin-coated beads the size of virions [153]. This suggested that nanchangmycin blocks the size-dependent endocytic internalization pathway required for the uptake of flaviviruses. While the target of the small molecule remains unknown, there is a connection between nanchangmycin and RNASEK and LY6E [153]. In human U2OS cells, nanchangmycin exhibited an IC_50_ of 158 nM against WNV-Kun infection; however, the IC_50_ dropped substantially to 62 nM and 7 nM in the presence of siRNAs targeting LY6E and RNASEK, respectively. The observation that LY6E and RNASEK depletion impacts nanchangmycin potency implies that nanchangmycin targets the same size-dependent endocytic pathway. Future studies on nanchangmycin mechanism will not only inform flavivirus biology but will also provide novel avenues for antiviral targeting of viral entry.

Endosomal acidification is required for entry of flaviviruses, at least in cell culture, and several drugs target this step in the virus entry pathway. Chloroquine, a lysosomotropic FDA-approved anti-malarial agent, has been extensively explored as an antiviral and detailed as an inhibitor of viral endocytosis [271,272]. Specifically, chloroquine and its analogs act as a weak base to prevent the acidification of endosomal compartments, a critical step in many virus life cycles, which explains its demonstrated efficacy in blocking flavivirus infection [235,236,237,238,273,274,275,276,277]. Though endosomal acidification is traditionally considered in the context of viral envelope fusion, it is likely that treatment with these inhibitors can impact additional steps of viral infection [278,279]. Hydroxychloroquine has also been described to both activate innate immune machinery during DENV infection and inhibit the ZIKV NS2B-NS3 protease [243,280]. However, it is clear that treatment with high levels of these drugs can present pathological consequences [281,282,283,284], and recent studies with SARS-CoV-2 highlight the fact that entry in vivo can be distinct from studies in cancer cell lines [230,285,286]. Chloroquine also showed limited efficacy in clinical trials for DENV-induced disease, failing to reduce the duration of the disease course and viremia [287,288]. Alternative structural analogs of chloroquine, such as amodiaquine, may be more successful down the line [224,225,226].

Bafilomycin A1, a macrolide antibiotic, has also shown efficacy in preventing acid-dependent steps of flavivirus endocytosis [155,221,231,232,233,289,290], albeit through differing mechanisms. Bafilomycin A1 inhibits the V-ATPase, making it a useful tool [198]. However, inhibiting this complex results in a diverse array of defects and toxicities [291,292,293,294,295,296]. Isoform selective targeting may bypass some of the toxicities given the possibility that only particular forms of the complex may be important for early steps of virus-dependent CME. 

Given the essential requirement of CME across diverse physiological processes (e.g., [148,297,298]), it would be transformative to identify antivirals that target a more selective form of CME. Currently, nanchangmycin is the only small molecule implicated in targeting this selective form of CME. However, future studies are essential to determine if indeed this candidate is active in animals.

## 5. Summary

The development of broad-spectrum antiviral therapeutics is essential to ameliorate the public health burden of mosquito-borne flavivirus infection. Virus entry has been successfully targeted in other viral infections. A common problem in targeting entry, however, is that mechanisms of entry often exhibit cell type-, virus-, serotype-, or strain-dependent behavior. An ideal scenario is the identification of a dependence unifying flaviviruses, or at least subgroups of flaviviruses. Interestingly, recent studies have begun to characterize a unique, specialized form of clathrin-mediated endocytosis with distinct requirements from canonical pathways and specific for larger cargo, such as virions. Though many details remain to be worked out, this pathway mediating virus endocytosis could potentially represent a non-essential host pathway that is necessary for viral infection. Most notably, the bacterial product nanchangmycin may target size-dependent endocytosis. If this specialized pathway is truly unique to flavivirus or general viral infection or has minor roles in normal cell biology, selective targeting of the pathway should minimize adverse side effects and promote clinical utility. Future studies are required to define this pathway and characterize this new antiviral. In addition, considering the pan-antiviral potential of the drug, as evidenced by its broad inhibition of viral infection including that of SARS-CoV-2 [230], continuing efforts towards development of this class of antivirals is essential.

## Figures and Tables

**Table 1:** Summary table highlighting antivirals that target or may target viral endocytosis. EC_50_ and IC_50_ correspond to the half-maximal effective and inhibitory concentrations, respectively. Each EC_50_ or IC_50_ value is associated with a specific virus, assay, and reference.

Name	Mechanism of Action	EC_50_	IC_50_	Tested Against	Assay	References
Amodiaquine	Inhibits endosomal acidification Other functions?	n/a	2.28 μM	ZIKV	Plaque	[224]
		3.07 ± 0.36 μM	n/a	ZIKV	MTT	[225]
		4.40 ± 0.51 μM	n/a		Plaque	
		7–11 μM	n/a	DENV-2	Replicon	[226]
		2.69 ± 0.40 μM	n/a		Plaque	
		17.67 ± 1.74 μM	n/a	DENV-4	Replicon	
		8–15 μM	n/a	WNV		
Apilimod *	Inhibition of endosomal trafficking via PI(3)P-5-kinase	n/a	9–136 nM	EBOV	ELISA	[227]
		n/a	10–140 nM	MARV		
		n/a	40 nM	LASV	Pseudovirus	[228]
		n/a	30 nM	EBOV		
		n/a	50 nM	EBOV	Pseudovirus	[229]
		n/a	50 nM	SARS-CoV-2		
		n/a	10 nM	SARS-CoV-2	Focus-forming	
		3–7 nM	n/a	SARS-CoV-2	Infectivity	[230]
Bafilomycin A1	Inhibition of V-ATPase-mediated acidification	?	?	DENV-2, JEV, WNV, YFV, ZIKV	n/a	[155,193,221,231,232,233]
BP34610	Targets viral envelope protein	480 ± 60 nM	n/a	DENV-2	Plaque	[234]
Chloroquine	Inhibits endosomal acidification	n/a	1.7–4.2 μM	ZIKV	qRT-PCR	[235]
		n/a	10 μM	ZIKV	IF	[236]
		4.95 ± 0.47 μM	n/a	ZIKV	MTT	[225]
		5.11 ± 0.62 μM	n/a		Plaque	
		9.82–14.2 μM	n/a	ZIKV	Cell viability	[237]
		5–10 μM	n/a	ZIKV	MTT	[238]
		5.12 ± 0.66 μM	n/a		Plaque	
Chlorpromazine	Blocks binding of AP-2 to the plasma membrane, D2R antagonist	?	?	DENV, JEV, WNV, ZIKV	n/a	[156,164,166,168,171,175,211,212,213]
Dynasore	Inhibitor of the GTPase dynamin	?	?	DENV, JEV, ZIKV	n/a	[168,171,175,213,239]
Epigallocatechin gallate	?	n/a	7.0 μM	JEV	Plaque	[240]
		n/a	7.9 μM		Attachment	
		n/a	9.4 μM		Entry	
		21.4 μM	n/a	ZIKV	Focus-forming	[241]
		14.8 ± 2.6 μM	n/a	DENV-1	ELISA	[242]
		18.0 ± 1.0 μM	n/a	DENV-2		
		11.2 ± 1.7 μM	n/a	DENV-3		
		13.6 ± 0.0 μM	n/a	DENV-4		
Hydroxychloroquine	Activates host innate immunity, inhibitor of viral protease	n/a	10–13 μM	DENV-2	Fluorescent intensity	[243]
Isoquercitrin	?	n/a	10–15 μM	ZIKV	Plaque	[244]
Nanchangmycin	?	n/a	100–400 nM	ZIKV	Infectivity	[133]
		n/a	158 nM	WNV	Infectivity	[153]
Niclosamide	Prevents endosomal acidification	n/a	15 μM	DENV-1	Infectivity	[245]
		n/a	400 nM	DENV-2		
		n/a	1.6 μM	DENV-3		
		n/a	700 nM	ZIKV		
		n/a	220–280 nM	ZIKV	Focus-forming	[246]
		10 μM	n/a	DENV-2	Plaque	[247]
		480 ± 60 nM	n/a	ZIKV	Plaque-reduction	[248]
		550 ± 50 nM	n/a	DENV-2		
		540 ± 170 nM	n/a	WNV		
		840 ± 20 nM	n/a	YFV		
		1.02 ± 80 μM	n/a	JEV		
		5.80 μM	n/a	JEV	Plaque-reduction	[249]
Pitstops *	Block ligand association with clathrin terminal domain	?	?	CCHFV, HCMV, HBV, HIV	n/a	[250,251,252,253]
Prochlorperazine	Interferes with clathrin associated pathways, D2R antagonist	88 nM	n/a	DENV-2	Plaque	[220]
		137 nM	n/a			
TK1023	?	1.55–1.68 μM	n/a	ZIKV	Plaque	[254]
Tyrphostin A9	RTK inhibitor, inhibitor of AP-2 complexes?	n/a	1.4 μM	DENV-1	Infectivity	[245]
		n/a	400 nM	DENV-2		
		n/a	1.2 μM	DENV-3		
		n/a	300 nM	YFV		
		n/a	300 nM	ZIKV		

* No available data against mosquito-borne flaviviruses.
